# Multiparameter interferometric polarization-enhanced imaging differentiates carcinoma *in situ* from inflammation of the bladder: an *ex vivo* study

**DOI:** 10.1117/1.JBO.28.10.102907

**Published:** 2023-08-10

**Authors:** Shuang Chang, Giovanna A. Giannico, Ezekiel Haugen, Ali Jardaneh, Justin Baba, Anita Mahadevan-Jansen, Sam S. Chang, Audrey K. Bowden

**Affiliations:** a Vannderbilt University, Vanderbilt Biophotonics Center, Department of Biomedical Engineering, Nashville, Tennessee, United States; b Vanderbilt University Medical Center, Department of Pathology, Microbiology, and Immunology, Nashville, Tennessee, United States; c Vanderbilt University Medical Center, Department of Urology, Nashville, Tennessee, United States; d Vanderbilt University, Department of Electrical and Computer Engineering, Nashville, Tennessee, United States

**Keywords:** bladder cancer, inflammation, polarization, optical coherence tomography, carcinoma *in situ*

## Abstract

**Significance:**

Successful differentiation of carcinoma *in situ* (CIS) from inflammation in the bladder is key to preventing unnecessary biopsies and enabling accurate therapeutic decisions. Current standard-of-care diagnostic imaging techniques lack the specificity needed to differentiate these states, leading to false positives.

**Aim:**

We introduce multiparameter interferometric polarization-enhanced (MultiPIPE) imaging as a promising technology to improve the specificity of detection for better biopsy guidance and clinical outcomes.

**Approach:**

In this *ex vivo* study, we extract tissue attenuation-coefficient-based and birefringence-based parameters from MultiPIPE imaging data, collected with a bench-top system, to develop a classifier for the differentiation of benign and CIS tissues. We also analyze morphological features from second harmonic generation imaging and histology slides and perform imaging-to-morphology correlation analysis.

**Results:**

MultiPIPE enhances specificity to differentiate CIS from benign tissues by nearly 20% and reduces the false-positive rate by more than four-fold over clinical standards. We also show that the MultiPIPE measurements correlate well with changes in morphological features in histological assessments.

**Conclusions:**

The results of our study show the promise of MultiPIPE imaging to be used for better differentiation of bladder inflammation from flat tumors, leading to a fewer number of unnecessary procedures and shorter operating room (OR) time.

## 1 Introduction

The ability to demarcate malignant tissues from surrounding benign inflammation is critical to surgical planning,^1^^–^^5^ tumor resection,^6^^,^^7^ and therapeutic decisions^8^ in many clinical indications. Inadequate resection and unnecessary biopsy due to false positives or low detection confidence are costly consequences of the inability to differentiate tumor from inflammation, leading to an increased number of operating room visits, prolonged operating room time, increased morbidity, and, ultimately, increased financial burden on the healthcare system. In particular, urinary bladder cancer (UBC) is the most expensive cancer to treat per patient’s lifetime,^9^ in large part because its high recurrence rate (50% to 70%)^10^ demands frequent patient surveillance and biopsy (every 3 to 12 months).^11^ The close resemblance of inflammation to carcinoma *in situ* (CIS), a high-grade,^12^ flat tumor and the leading cause of recurrence in UBC,^13^^,^^14^ is a significant barrier to the detection and eradication of malignant tissue, as the current gold standard technology for bladder cancer surveillance, white light cystoscopy (WLC), has low sensitivity (62% to 84%) and specificity (43% to 93%) for consistently detecting CIS.^15^

Given the frequency of surveillance, non-ionizing, optical techniques are the preferred tools for bladder cancer surveillance. Clinically approved blue light cystoscopy (BLC) and narrowband imaging are established alternatives to WLC that improve the sensitivity of CIS detection through the visualization of absorption-specific contrast;^16^^,^^17^ however, both suffer from high false-positive rates (>30%)^18^^–^^21^ and poor specificity, leading to unnecessary biopsies.^22^ Photoacoustic imaging with bladder-cancer-cell-line-targeted gold nanorods was recently shown to detect cancerous lesions smaller than 0.5 mm; however, this demonstration was limited to animal models and does not readily combine with cystoscopy imaging.^23^ Moreover, the safety of the ultrasound-assisted shaking needed to achieve uniform delivery of nanorods in the bladder has yet to be demonstrated. Microscopic imaging technologies that enable imaging below the mucosal surface have shown promise as emerging adjuvants for cystoscopy, including confocal laser endomicroscopy (CLE) and interference-based optical coherence tomography (OCT). Both imaging modalities can integrate with clinical cystoscopy tools and create real-time “optical biopsies” that allow for visualization of subsurface structures to assist diagnosis.^24^^,^^25^ However, CLE, similar to the standard-of-care tools, relies on visual assessment, leading to challenges in interobserver agreement (63.6%).^26^ Although CLE achieves 75% sensitivity and 64% specificity among experienced clinicians, only 46% sensitivity and 74% specificity were achieved when reviewed by non-experienced clinicians. In addition, CLE does not image beyond the first layer of the bladder (up to 65  μm imaging depth below the surface) and may thus fail in conditions of recurrent carcinoma under post-resection scars. Moreover, CLE requires a fluorescent contrast agent, which may cause adverse reaction through intravenous injection or require extended preparation time if administered through intravesical instillation, limiting its usage to the operating room.

*In vivo* OCT exhibits better sensitivity (75% to 100%) and specificity (78% to 90%) than WLC and CLE,^27^^,^^28^ and it improves the false-positive rate when used alongside existing clinical tools such as BLC.^18^^,^^29^ The key to OCT’s effectiveness is its ability to directly visualize subsurface features three layers deep [i.e., down to the muscularis propria (MP) layer] that match histologically relevant tumor-specific characteristics^30^^,^^31^ that can only be visualized in cross-section, such as denuding of the urothelium from the lamina propria (LP) and the invasion of cancer into the MP. However, conventional OCT images are strictly based on back-reflected light intensity and cannot sufficiently contrast CIS from inflammation, as both conditions present with decreased intensity in the LP and blurring of the characteristic border between the urothelium and LP.^6^

Collagen, which is abundant in the LP, exhibits birefringence that can be probed with polarized light,^32^ and the loss of birefringence has been recognized as a hallmark of cancer in many tissue types.^33^^,^^34^ Structural disorder associated with cancer development leads to reorganization, degradation, or excessive accumulation of collagen, which additionally has a depolarizing effect.^35^^,^^36^ Recently, we and others have shown that OCT with polarized light can visualize differences between CIS and inflamed bladder tissues.^37^^,^^38^ As a first step toward achieving the specificity necessary to avoid unnecessary biopsies, we introduce multiparameter interferometric polarization-enhanced (MultiPIPE) imaging as a new strategy to reliably differentiate CIS from inflammation, thereby reducing the number of false positive findings. MultiPIPE imaging converts micrometer-resolution, cross-sectional structural images obtained with polarization-sensitive OCT into quantitative biomarkers that effectively differentiate inflamed versus CIS tissue and benign versus CIS tissue with high specificity (92% and 95%, respectively). As such, MultiPIPE imaging is a compelling technology for integration into clinical workflows for UBC surveillance; moreover, the collagen-induced changes that it measures are common in other epithelial cancers (e.g., skin, stomach, and colon)^39^^–^^41^ and are relevant for several non-cancerous conditions (e.g., skin wounds, coronary plaque, and retinal disease),^42^^–^^44^ suggesting the potential for use of MultiPIPE imaging in broader biomedical research.

## 2 Materials and Methods

### 2.1 Patient Recruitment

**Fig. 1:**
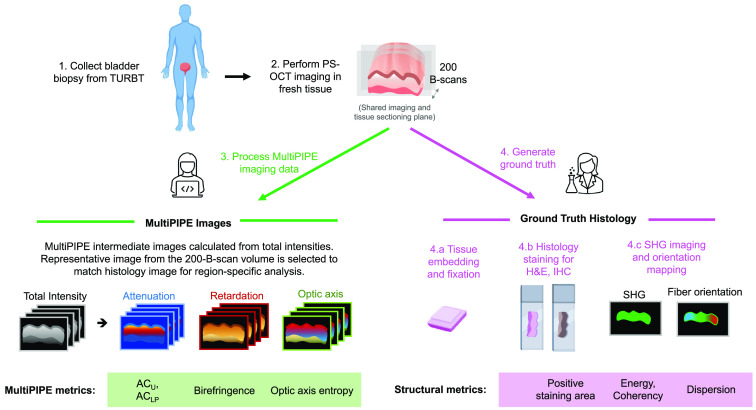
MultiPIPE analysis workflow. Fresh bladder biopsy samples were collected and imaged with PS-OCT within 1 h of resection. Following imaging, tissues were embedded and sectioned along the same or a parallel cross-sectional plane to that used for imaging. Fixed tissue slides underwent H&E and IHC staining of collagen type I and SHG imaging. MultiPIPE imaging data comprise cross-sectional intensity, attenuation, retardation, and optic axis images. Once paired with histology, quantitative measures of the regional attenuation coefficient (${\mathrm{AC}}_{U}$ and ${\mathrm{AC}}_{\mathrm{LP}}$), birefringence, and optic axis entropy were extracted from the MultiPIPE dataset.

We obtained benign and cancerous bladder biopsy samples from 27 patients undergoing transurethral resection of bladder tumor at the Vanderbilt University Medical Center after diagnosis with bladder cancer or suspicious bladder lesions based on standard-of-care methods, including WLC. The study was approved by the VUMC Internal Review Board (IRB# 191337). From each patient, two biopsies were obtained for research purposes: one biopsy of tissue suspected as diseased (papillary tumor or CIS) and one of tissue suspected as normal. In total, we collected 7 CIS, 18 inflamed, and 14 normal tissues. Thirteen tissue samples were identified as papillary tumors and therefore excluded from our analysis. Specimens used in this study were collected and analyzed following the steps shown in Fig. 1.

### 2.2 Sample Preparation and Imaging

After resection, tissues were placed in containers filled with saline, transferred to a small petri dish, and immersed in saline, where they remained during imaging. Imaging of tissues was performed within 60 min of resection to minimize degradation. We used a Telesto series polarization-sensitive OCT imaging system (TEL220PSC2, Thorlabs, Inc.) to acquire MultiPIPE data by sending circularly polarized light to the tissue samples. An immersion-type Z-spacer (OCT-IMM3, Thorlabs, Inc.) was attached to the objective for water-immersion imaging of tissue samples. The Z-spacer allows for imaging at a close distance to the tissue surface while reducing the strong back reflections. The axial and lateral resolutions of the system are 5.5 and 13  μm, respectively. For each tissue sample, we obtained a volumetric dataset by sampling a 4-mm by 1-mm en face area with 200 B-scans (cross-sectional image) per volume and 1024 A-scans per B-scan, using the polarization-imaging mode.

### 2.3 Histology Preparation and Staining

Immediately after imaging, the tissues were fixed in 10% neutral buffered formalin (Thermo Fisher Scientific). To facilitate pairing histology with MultiPIPE data, we performed special embedding, in which the orientation of the tissue surface was orthogonal to the wax surface, enabling cross-sectional tissue sectioning. Tissues were sectioned into six 5-μm cross-sections following the standard routine, in the same direction as OCT B-scans. A subset of the tissue samples was stained with hematoxylin and eosin (H&E). H&E images were used as ground truth for tissue-type determination and reviewed for indication of inflammatory response. The remaining subset of tissue samples underwent immunohistochemistry (IHC) staining to visualize the distribution of collagen structures. Collagen type I was used in the IHC staining because it is the most abundant type of collagen in the LP and it has been extensively studied in relation to bladder cancer and other cancers.^31^^,^^45^^,^^46^ To perform IHC, tissue slides were placed on the Leica Bond Max IHC Stainer and deparaffinized. Heat-induced antigen retrieval was performed on the Bond Max using epitope retrieval 1 solution for 20 min. Slides were incubated with anti-collagen I (Cat. #ab138492, abcam, Cambridge, Massachusetts) for 1 h at a 1:500 dilution, followed by dehydration, clearing, and coverslipping. All slides were digitized with a 20× brightfield slide scanner (Leica SCN400) and then imported to QuPath, an open-source software for digital pathology image analysis.^47^ All stained slides from a sample (H&E and IHC staining) were sent to the surgical pathologist for tissue type confirmation.

### 2.4 MultiPIPE Dataset Analysis

#### 2.4.1 Co-registration with histology

To compare the imaging data to histology (ground truth) and to accurately assess region-specific parameters, we manually matched MultiPIPE intensity images to histology slides. One image per volume was manually selected. Features from the top two layers of the total intensity image (i.e., the outline of the luminal surface and the structural features such as sites of inflammation sites, the presence of von Brunn’s nest, and urothelium thickness) were used to enable the pairing. The tissue surface and appearance in the intensity images were determined using Otsu’s thresholding method^48^ and were matched with the histology slide tissue surface and structures up to 500  μm below the surface. Inflammation sites, which contain lymphocyte aggregates or diffuse lymphocyte infiltration, appeared as low-intensity regions in the LP layer of the intensity image. Both inflammation sites and benign invaginations are distinct structures that were useful for pairing.

#### 2.4.2 Segmentation and attenuation coefficient analysis

Unless otherwise described, all processing and analyses were implemented in MATLAB R2021a (Mathworks, Inc.). MultiPIPE intermediate images were created for direct visualization of the differences in tissue content. Researchers were blinded to the tissue-type information during the analysis. The centermost 2-mm-wide region of the image chosen for co-registration was used for a given MultiPIPE dataset in data analysis.

**Fig. 2:**
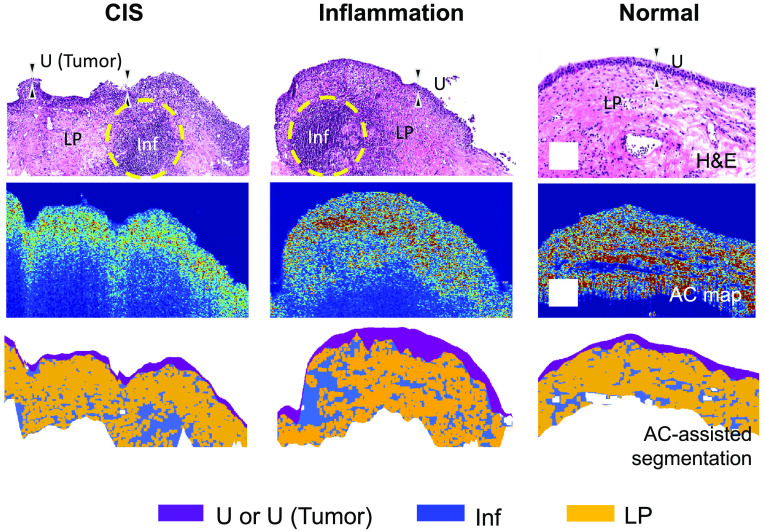
AC-assisted segmentation procedure to facilitate MultiPIPE analysis. H&E, attenuation image, and resulting AC-assisted segmentation map of representative CIS, inflammation, and normal samples. The color-coded AC-assisted segmentation map shows the urothelium (purple), inflammation regions in the LP (blue), and the normal LP (yellow). Dashed yellow lines indicate regions of inflammation in the H&E slides. U, urothelium; Inf, inflammation; LP, lamina propria. Scale bar: 200  μm.

The attenuation coefficient (AC) measures the rate of light attenuation in tissue and is governed by the properties of the tissue.^49^ Thus, accurate estimation of AC can facilitate differentiation of dissimilar tissues or tissue layers. To segment the image into regions for analysis, the AC at each image voxel was computed following a depth-resolved method,^50^^,^^51^ and the cross-sectional images of the AC were generated for each tissue specimen. The resulting AC images allowed for more meaningful visualization of sub-surface features with better contrast. We adopted a previously demonstrated automated AC-assisted layer detection algorithm to segment and characterize tissue regions as urothelium,^52^ LP, or inflammation, as shown in Fig. 2. We computed the average AC value from the urothelium and the entire LP (including inflammation sites) region for statistical analysis.

#### 2.4.3 Analysis of tissue birefringence

**Fig. 3:**
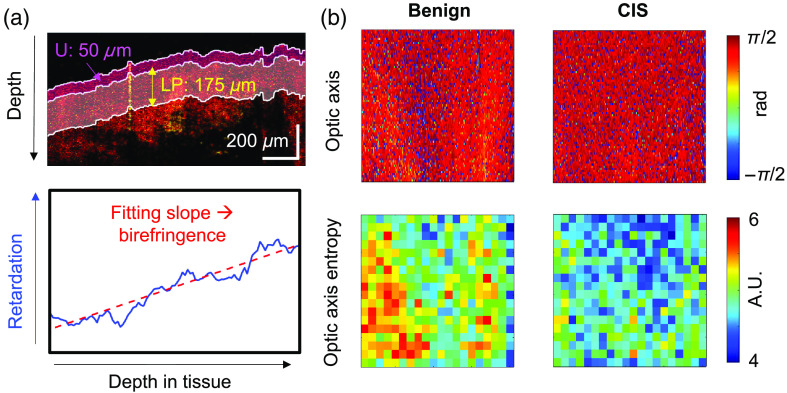
Birefringence calculation and OA entropy calculation from the LP region. (a) Birefringence calculation from the LP region. The U and LP regions are naively segmented in the retardation map at 50 and 225  μm below the tissue surface, respectively. For each B-scan, the depth-dependent retardation measurements are averaged laterally within the LP region and a linear regression is performed to the retardation versus depth plot. The fitting slope is then used to determine the birefringence of the LP layer. (b) OA entropy calculation from the LP region. The LP region is defined the same way in the OA mapping. For the OA measurements in the LP region (top row), entropy maps (bottom row) are generated using a unit area of 4 pix (10  μm depth) by 20 pix (78  μm lateral). From each OA entropy map, a mean entropy value is determined and used in statistical analysis. In the example of benign and CIS OA mappings, the calculated entropy is higher in the benign than the CIS sample, suggesting greater changes in the OA with depth for the benign tissue.

Birefringence, Δn, is determined from the linear relationship between phase retardation versus depth in the equation below:

δ=2π Δn z/λ, (1)

where λ is the center wavelength of the incident light, z is the round-trip distance of light traveled in tissue, and δ is the cumulative phase retardation. To determine the birefringence of the LP region of the bladder, we first performed a simple segmentation, for which we assumed that 50  μm below the tissue surface is the urothelium region and 175  μm from the bottom of the urothelium is the LP region. We assumed that most of the tissue birefringence was contributed by the collagen in the LP layer and that the contribution from the urothelium was negligible, as shown by a previous study.^37^ From the “LP” region, we computed the sample birefringence. After flattening the image by aligning the surface position across different lateral locations, we averaged the retardation laterally over the entire region of interest (ROI), and the birefringence was extracted from the slope of a line fitting the retardation versus depth. An example of the fitted line is shown in Fig. 3(a).

#### 2.4.4 Analysis of OA entropy

In biological tissue imaging, the orientation of a fibrous structure can be characterized by the orientation of the optic axis.^53^ For tissues that have a high collagen content (i.e., the tissue is birefringent), the measured OA rotates with depth. In this study, we measured the cumulative OA. For highly birefringent tissues, the cumulative OA varies along depth, whereas for tissues with no birefringence, the cumulative OA experiences minimal variation. The degree of the variation, and thus the heterogeneity, or entropy of the OA measurements within a unit area, has been used to describe the arrangement of collagen fibers. Entropy measurements allow for easier interpretation and analysis than the original OA mapping.^42^ The OA entropy, H, is calculated with a histogram method and is defined as

$$H=-\sum p_{i}\;\log_{2}(p_{i}),$$ (2)

where i is a bin representing an OA angle in the measured OA image and $p_{i}$ is the number of occurrences of OA angle per analyzed area. In a unit area with OA angles that exhibit a large magnitude of variations, the entropy is high, and for an area with consistent OA measurements, the entropy is low, as shown in Fig. 3(b). The unit area used in the study is 10  μm (depth) by 78  μm (laterally), which takes into the consideration the resolution of the imaging system and the size of the B-scan.

### 2.5 Second Harmonic Generation Imaging and Analysis

Second harmonic generation (SHG) microscopy is a powerful tool to visualize the organization of type I collagen fibers in tissue, and it has been utilized to study a variety of pathologies, including cancer development.^54^ SHG occurs when two near-infrared photons simultaneously interact with a non-centrosymmetric structure (e.g., collagen fibrils) and release a photon with the summed energy of the initial photons.^55^^,^^56^ SHG imaging was performed using a multimodal nonlinear microscope system on H&E stained 5  μm-section slides, and thus SHG images were co-registered with H&E and MultiPIPE images.^57^ A femtosecond laser source centered at 1040 nm (Spectra Physics Insight DS+) was utilized to induce SHG at 520 nm (i.e., twice the energy of the incident beam). The beam was directed to a galvanometric mirror pair and relayed via a 4× magnifier (Thorlabs SL50-2P and TL200-2P2) to a water immersion objective lens (Olympus XLUMPFLN 20× 1.0 NA). Images were acquired over a 648×648  μm field-of-view (FOV) at ∼0.3  μm/pixel, with 7  μs/pixel dwell times and ∼10  mW average power at the sample. SHG was collected using a green filter (525±25  nm; Semrock) and epi-detected with a photomultiplier tube (Thorlabs GaAsP amplified PMT).

**Fig. 4:**
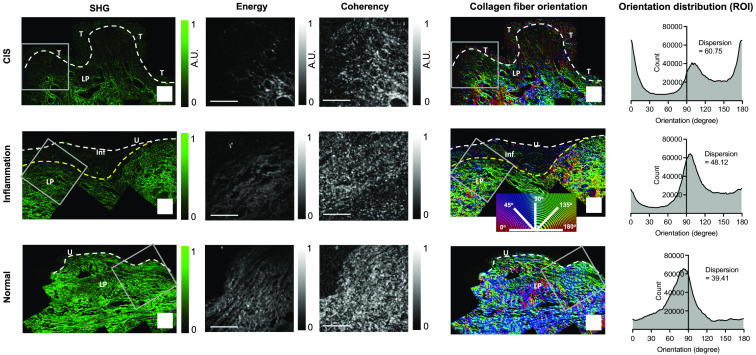
SHG-derived maps of computed energy, computed coherency, and collagen fiber orientation. Columns 1 to 3: The energy and coherency maps derived from SHG images of representative CIS, inflammation, and normal samples indicate the strength of collagen fiber alignment. Gray boxes indicate ROIs from which energy and coherency maps are plotted. White dashed lines indicate the location of the urothelial basement membrane, and the yellow dashed line denotes the inflamed region in the inflamed sample. Normal samples have the highest energy and coherency; CIS samples have the lowest. Column 4–5: Maps of collagen fiber orientation angle (left) and plots of its distribution (right) as derived from the representative areas in the gray-boxed regions. White dashed lines indicate the location of the urothelial basement membrane, and the yellow dashed line denotes the inflamed region in the inflamed sample. Here, the distribution of collagen orientation angles shows strong alignment in the normal sample but is more dispersed in the CIS sample. The inflamed sample also shows relatively strong alignment but slightly more dispersion than the normal sample. These differences can be quantified via the orientation angle dispersion. T, tumor; LP, lamina propria; U, urothelium; inf, inflammation. Scale bar: 200  μm.

Tissue regions above the MP were imaged as a sequence of FOVs with a small overlap. To visualize collagen fiber organization across the tissue section, SHG images with overlapping FOVs were stitched using MosaicJ in FIJI (Version 2.3.0).^58^^,^^59^ Representative stitched SHG images are shown in Fig. 4. The local orientations of collagen fibers were quantified using OrientationJ (Version 2.0.5) with a 6-pixel (∼1.8  μm) Gaussian window structure tensor and color-coded on top of the SHG image as shown in Fig. 4.^60^^,^^61^

The pixel-wise isotropic properties (energy and coherency) of the collagen structure were quantified with the same structure tensor in OrientationJ. Energy and coherency maps were computed for each SHG image, as shown in Fig. 4. The energy is equal to the trace of the structure tensor, with high energy values suggesting more aligned structures and low energy values suggesting more isotropic structures. Coherency is a normalized parameter that is given by the difference divided by the sum of the maximum and minimum tensor eigen values.^61^ Coherency values fall in the range of 0 to 1, with 0 indicating isotropic regions and 1 being highly oriented regions.

Raw, unstitched SHG images were analyzed in OrientationJ to extract the orientation angle distributions. The output orientation counts were imported to MATLAB to compute a normalized distribution. A kernel probability distribution was then fit to the distribution plot of orientation angles, and the dispersion was calculated as the standard deviation of the fitted distribution. Example orientation angle distributions (shown as a scaled ratio) for CIS, inflammation, and normal tissues are shown in Fig. 4.

### 2.6 Histology Staining Analysis

Digitized histology slides were imported into QuPath, and regions just below the lumen surface of the tissue of size 300  μm by 300  μm were selected for analysis. All selected regions are above the MP to restrict our analysis to superficial layers of the bladder wall (the urothelium and the LP region). The positively stained areas from IHC images were extracted using an optimized setting in the software (resolution=0.5  μm per pixel, threshold = 0.25, smoothing sigma = 0), and the collagen density was calculated as a ratio of the positive staining regions over the entire tissue region. Researchers were blinded to the tissue type information during the measurement.

### 2.7 Tissue Type Confirmation

One surgical pathologist who was blinded to the MultiPIPE data reviewed the histology slides (H&E and IHC collagen) for all samples and performed tissue-type assessment, grading, and staging. Tissue types determined at the time of histology, including normal, inflammation, and cancer, were used in the study for statistical analysis. Because there is a discrepancy between the tissue-type assessment by clinicians at the time of tissue collection and by pathologists upon histological review, both “normal-looking” and “disease-looking” tissues at collection were occasionally found to be inflamed samples. We first excluded all papillary tumors from the study because we limited our analysis to fresh samples of benign tissues and CIS tumors. For a tissue sample that exhibited thermal damages, we only included it in clinical sensitivity and specificity calculation, but we excluded it from MultiPIPE metric analysis and histology imaging analysis, because thermal damages drastically alter the optical properties of tissue. A scar tissue sample (without LP) was also excluded from all analyses except in clinical sensitivity and specificity calculation.

### 2.8 Statistical Analysis

Statistical analysis of MultiPIPE parameters between pairs of study groups was based on the Mann-Whitney U test. Statistical significance was calculated in Prism (GraphPad Software, Version 9.0.0). Descriptive statistics [mean and 95% confidence interval (CI) of the mean] were computed with Prism. Receiver operating characteristic (ROC) curve analysis was performed in Prism to evaluate the ability of each MultiPIPE parameter to differentiate CIS from inflammation or CIS from benign tissues. The areas under the curve (AUCs) with 95% confidence bounds are reported for each ROC curve. To build a multi-parameter classifier, we developed a least absolute shrinkage and selection operator (LASSO) logistic regression model in MATLAB, with all MultiPIPE parameters as predictor variables and using histologically confirmed tissue type as the ground truth classification.^62^ We used threefold cross-validation to determine the optimal model coefficient by locating the LASSO tuning parameter, lambda, with the minimum cross-validation error. The lambda with the minimum deviance was chosen to fit the final logistic model. ROC curve analyses were carried out to evaluate the effectiveness of differentiation. Assuming the sample distribution of the study is approximately the population distribution, pointwise CIs were computed using 1000 bootstrap replicas.

## 3 Results and Discussion

**Fig. 5:**
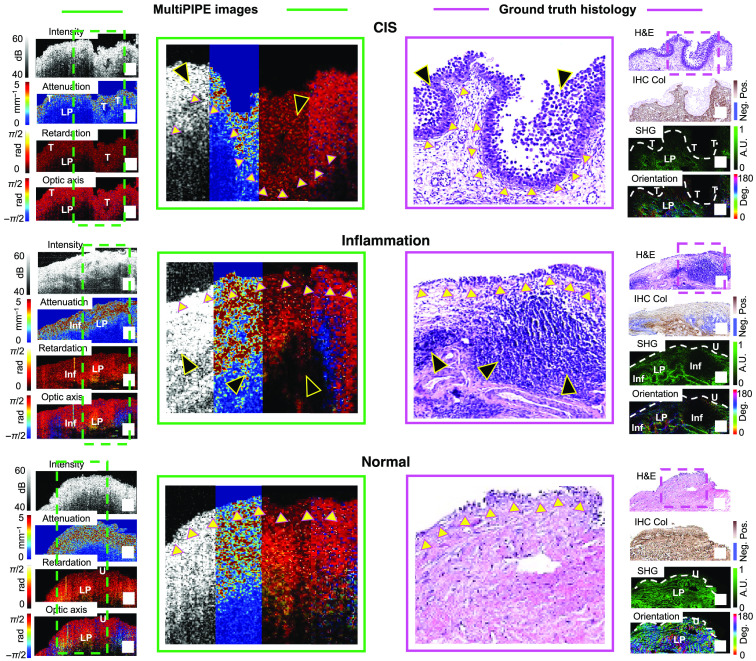
Visual analysis of MultiPIPE data captures differences in CIS and inflammation visible in histology. Example cases of CIS (top), inflammation (middle), and normal (bottom) samples. Left column: intensity, attenuation, retardation, and optic axis images. The attenuation image shows higher attenuation in the LP region of the inflamed sample than in CIS. The attenuation image shows the highest attenuation in the LP region of the normal sample. In the retardation and optic axis images, the CIS sample shows less variation in depth than in inflammation, and the normal sample shows the largest variation in depth. Dashed boxes show zoomed-in areas for each image that appear in the middle column. Middle column: zoomed portion of MultiPIPE and H&E images indicated by green and purple dashed boxes, respectively. Small yellow triangles point at the basement membrane of the urothelium, and large black triangles point at tumorous (top image) and inflammatory (bottom image) regions, respectively. Right column: H&E, IHC, SHG, and SHG-based collagen fiber orientation images. Comparing the SHG and orientation maps of CIS and inflammation, the LP regions near the CIS tumor show pronounced degradation of the collagen fibers evidenced as significantly diminished SHG signal and loss of directionality; similarly, the LP of the inflamed sample shows localized thinning of the collagen meshwork and changes of fiber orientation surrounding the lymphoid aggregates. The SHG and orientation maps of the normal sample show strong, uniform SHG signal and well-maintained directionality. White dashed lines in the SHG images outline the basement membrane of the urothelium. T, tumor; LP, lamina propria; U, urothelium; inf, inflammation. Scale bar: 200  μm.

The standard outputs of PS-OCT are cross-sectional images of backscattered light intensity incident at two orthogonal polarization states; the sum of these images yields the total backscattered intensity and resembles a conventional (unpolarized) OCT image. MultiPIPE analysis produces three intermediate images that visualize light attenuation, light retardation, and optic axis orientation; the latter two characterize the tissue’s response to polarized light. These intermediate images are then further processed to extract, respectively, three final parameters of interest: regional AC for the urothelium and LP (${\mathrm{AC}}_{U}$ and ${\mathrm{AC}}_{\mathrm{LP}}$), regional birefringence in the LP, and regional optic axis (OA) entropy in the LP. The emphasis on changes in the LP stems from observations of the image regions exhibiting large distinctions between CIS, inflammation, and normal tissue types. Representative samples appear in Fig. 5. Notably, the intensity in the LP region of CIS tissue is lower than in benign tissues (inflamed and normal); the CIS sample also exhibits smaller variations in attenuation (i.e., lower AC), retardation (i.e., lower birefringence), and optic axis (i.e., lower OE) as a function of depth. The AC and the depth-dependent variations in retardation and optic axis are largest in the normal sample. In inflamed tissues, the localized reductions in the AC and in the variations of the retardation and optic axis coincide with areas of inflammatory response (e.g., lymphoid aggregates) that are visible on histology.

The observed retardation and optic-axis changes visible in the MultiPIPE images correlate with morphological changes revealed by SHG imaging, which are largely dominated by the presence of type 1 collagen fibers. The CIS sample has a diminished collagen signal, whereas the inflamed sample maintains signal in the LP regions surrounding lymphoid aggregates, and the normal sample exhibits a strong, uniform collagen signal. The computed energy and coherency maps from the SHG images indicate how isotropically (i.e., low energy and coherency) or aligned (i.e., high energy and coherency) the fiber structures are organized. The CIS sample shows lower SHG intensity, energy, and coherency compared with the inflamed sample. Maps and distribution plots of the collagen fiber orientation angle in the LP generated from SHG data reveal notable differences that are characterized by their dispersion: CIS and inflammation show greater heterogeneity in fiber orientation angles (i.e., high dispersion), whereas the normal sample has more aligned fibers (i.e., low dispersion) and a dominant orientation angle. Note that, because the SHG image plane (i.e., the histology image plane) was the same or parallel to the MultiPIPE intensity image plane, the fiber orientation was analyzed within the cross-sectional plane and therefore the SHG orientation angles were not expected to correspond to the OA orientation measurement.

**Fig. 6:**
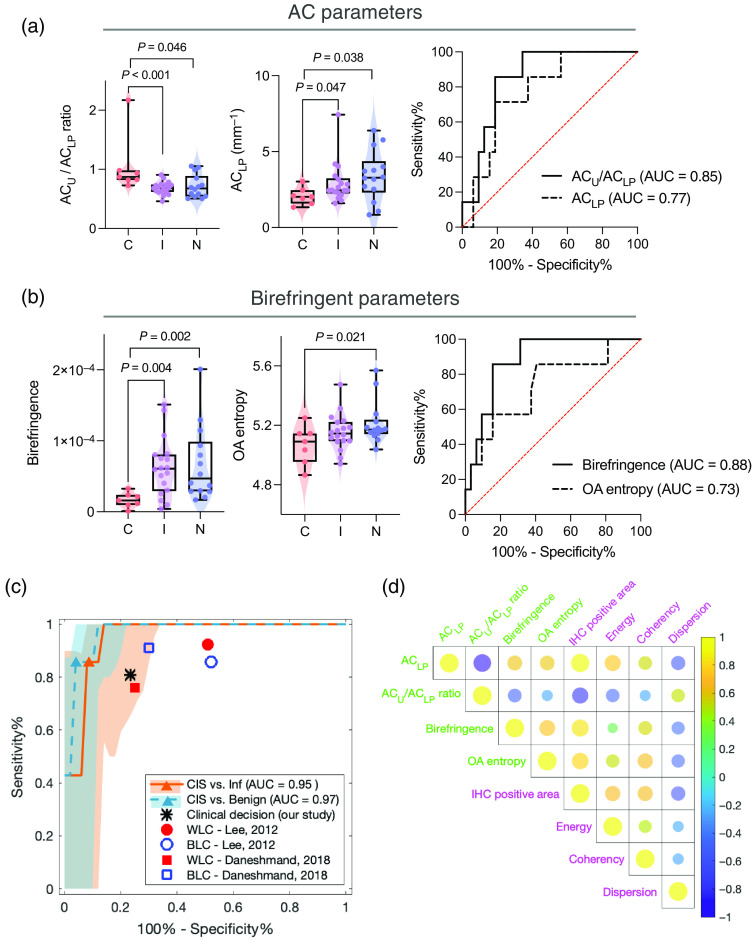
Metrics extracted from MultiPIPE data allow for successful differentiation of CIS from benign samples with high sensitivity and specificity. (a) Analysis of AC metrics. Violin and box plot of (left) urothelium to LP AC ratio and (middle) AC value measured from the LP region in CIS, inflammation, and normal samples; (right) ROC curves to differentiate CIS from benign samples (inflammation and normal) using the two AC metrics. AUC values are reported for both. (b) Analysis of birefringent metrics. Violin and box plot of (left) birefringence and (middle) OA entropy measured from the LP region of CIS, inflammation, and normal tissues; (right) ROC curves to differentiate CIS from benign samples using the two birefringent metrics. AUC values are reported for both. (c) ROC curves are shown of multiparameter logistic regression model used for the classification of CIS versus inflammation and CIS versus benign samples. ROC curves are graphed with CIs determined using the bootstrap method ($N_{\text{boot}}=1000$). Orange and teal triangles show the locations on the ROC curves where the sensitivity and specificity measurements were obtained. (d) Correlation plot is shown to explore the correlations in MultiPIPE parameters (LP AC, U/LP AC, birefringence, OA entropy) and biological features (IHC positive staining area, SHG energy, and SHG orientation distribution). The colors and sizes of the circle indicate the degree of correlation, with yellow being strong-positively correlated and blue being strong-negatively correlated.

**Fig. 7:**
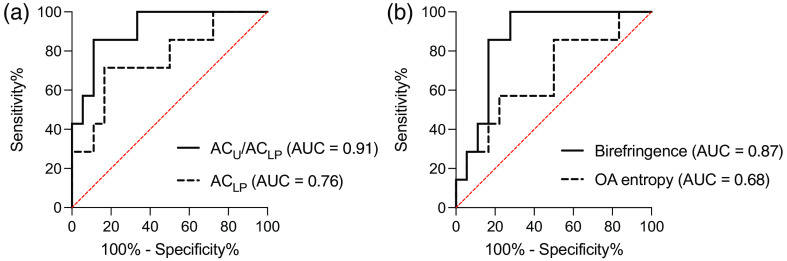
ROC curves of AC and birefringent metrics for CIS versus inflammation. (a) Analysis of AC metrics. ROC curves to differentiate CIS from inflammation using ${\mathrm{AC}}_{U}/{\mathrm{AC}}_{\mathrm{LP}}$ and ${\mathrm{AC}}_{\mathrm{LP}}$ metrics are shown; these achieved AUC values (with 95% CI) of 0.91 (0.80 to 1.00) and 0.76 (0.54 to 0.98), respectively. (b) Analysis of birefringent metrics. ROC curves to differentiate CIS from inflammation samples with birefringence and OA entropy. AUC values achieved (with 95% CI) are 0.87 (0.72 to 1.00) and 0.68 (0.43 to 0.92), respectively.

Figure 6 summarizes the results of the quantitative metrics derived from MultiPIPE analysis after segmentation of the intermediate images, as described in Secs. 2.4.2 and 2.4.3. The mean values of the ${\mathrm{AC}}_{\mathrm{LP}}$ for CIS, inflammation, and normal samples were 2.06 [95% CI $({\mathrm{CI}}_{95})=1.54$ to 2.57], 2.92 (${\mathrm{CI}}_{95}$: 2.26 to 3.58), and 3.40 (${\mathrm{CI}}_{95}$: 2.44 to 4.36) ${\mathrm{mm}}^{-1}$, respectively. Statistically significant differences were obtained both for CIS versus inflammation (p<0.05) and for CIS versus normal (p<0.05). Statistical significance between CIS and inflamed tissue (p<0.001) as well as CIS and normal (p<0.05) was also achieved when comparing the ${\mathrm{AC}}_{U}/{\mathrm{AC}}_{\mathrm{LP}}$ ratios from the CIS (1.05; ${\mathrm{CI}}_{95}$: 0.59 to 1.51), inflamed (0.69; ${\mathrm{CI}}_{95}$: 0.63 to 0.74), and normal samples (0.71; ${\mathrm{CI}}_{95}$: 0.60 to 0.83). These results support the utility of AC-based information for differentiating clinical pathology, consistent with past work demonstrating the use of AC information in a broad range of clinical applications, including tumor detection and grading in urology.^63^ Importantly, significance was not found when using the conventional OCT metrics such as the intensity of the LP region or the ratio of intensities from the urothelium and the LP, consistent with past findings.^7^ The receiver operating characteristic (ROC) curves in Fig. 6(a) quantify the ability of ${\mathrm{AC}}_{U}/{\mathrm{AC}}_{\mathrm{LP}}$ and ${\mathrm{AC}}_{\mathrm{LP}}$ metrics to differentiate CIS from benign samples. The obtained AUCs are 0.77 (${\mathrm{CI}}_{95}$: 0.61 to 0.94) and 0.85 (${\mathrm{CI}}_{95}$: 0.73 to 0.98). The result for CIS versus inflammation is reported in Fig. 7(a).

Changes in collagen structures during early-stage cancer development manifest as changes in birefringence^33^^,^^64^ and have been reported in studies of the bladder and many other tissue types.^31^^,^^65^^–^^67^ We calculated the mean birefringence (with 95% CI) measured from CIS, inflammation, and normal LP to be $1.66\times 10^{-5}$ (${\mathrm{CI}}_{95}$: $6.97\times 10^{-6}$ to $2.62\times 10^{-5}$), $6.27\times 10^{-5}$ (${\mathrm{CI}}_{95}$: $4.22\times 10^{-5}$ to $8.32\times 10^{-5}$), and $6.73\times 10^{-5}$ (${\mathrm{CI}}_{95}$: $3.70\times 10^{-5}$ to $9.75\times 10^{-5}$), respectively. Strong statistically significant differences exist both for CIS versus inflammation (p<0.01) and for CIS versus normal (p<0.01). The mean entropy of the OA, a measure of the disorder of fiber alignment, in the LP region of CIS, inflammation, and normal samples was calculated to be 5.07 (${\mathrm{CI}}_{95}$: 4.95 to 5.19), 5.16 (${\mathrm{CI}}_{95}$: 5.10 to 5.22), and 5.21 (${\mathrm{CI}}_{95}$: 5.13 to 5.30), respectively. Statistically significant differences were found between CIS and normal samples (p<0.05) but not for CIS and inflammation (p=0.198), suggesting a progression of order to disorder from normal to CIS samples. ROC curves to differentiate CIS from benign samples (inflammation and normal combined) yielded AUC values of 0.88 (${\mathrm{CI}}_{95}$: 0.77 to 0.99) and 0.73 (${\mathrm{CI}}_{95}$: 0.52 to 0.95) for birefringence and OA entropy, respectively, as shown in Fig. 6(b). The performance of these metrics to differentiate CIS from inflammation is presented in Fig. 7(b).

We developed a logistic regression model to classify samples (CIS versus inflammation and CIS versus Benign) using the four MultiPIPE metrics (Fig. 6). The ROC curve for the multiparameter model on CIS versus inflammation yielded an AUC value of 0.95 (${\mathrm{CI}}_{95}$: 0.71 to 1) and sensitivity and specificity values of 85.7% and 92.0%, respectively. The ROC curve for the multiparameter model on CIS versus benign samples yielded an AUC value of 0.97 (${\mathrm{CI}}_{95}$: 0.87 to 1) and sensitivity and specificity values of 85.7% and 95.0%, respectively. The sensitivity and specificity of the clinical decisions in this study were calculated using the tissue types suspected at the time of collection during the cystoscopy procedure as positive/negative test results and the final pathology results as the ground truth. Only 6 of 14 samples thought to be CIS upon collection were histologically confirmed as true CIS (false positive rate = 23.5%): one was determined to be fibrin tissue (excluded from the study), and the rest were inflammation. Among samples collected as normal controls (n=27), 26 were truly benign, and one was histologically confirmed as CIS. We excluded one normal sample from analysis due to thermal damage to the sample surface that occurred during the clinical procedure. For CIS versus benign samples, the sensitivity and specificity of the clinical decisions made in this study were 85.7% and 76.5%, respectively. The sensitivities determined by the classifier and clinical decisions are comparable, whereas the specificity of the MultiPIPE model for CIS versus benign samples is 18.5% higher than clinical decisions reported here and in other studies;^68^^,^^69^ MultiPIPE imaging also reduces false positives (false-positive rate = 5%) by more than fourfold.

Correlation analysis of the tissue feature metrics derived from morphological analysis of SHG and IHC images with the MultiPIPE metrics, shown in Fig. 6(d), reveals positive correlations between ${\mathrm{AC}}_{\mathrm{LP}}$, birefringence, and OA entropy with IHC positive staining area (i.e., the ratio of the region of positive staining for collagen I antibody to the total tissue region) and SHG energy and coherency; it also shows negative correlations with SHG orientation dispersion. Results for the AC ratio (${\mathrm{AC}}_{U}/{\mathrm{AC}}_{\mathrm{LP}}$) show the opposite findings, which is intuitive because it is proportional to the inverse of ${\mathrm{AC}}_{\mathrm{LP}}$. Among MultiPIPE metrics, strong positive correlations are found between ${\mathrm{AC}}_{\mathrm{LP}}$, birefringence, and OA entropy, and all three measurements show negative correlation with ${\mathrm{AC}}_{U}/{\mathrm{AC}}_{\mathrm{LP}}$. Among morphological metrics, SHG energy, coherency, and IHC positive staining area are positively correlated with each other but are negatively correlated with orientation dispersion.

**Fig. 8:**
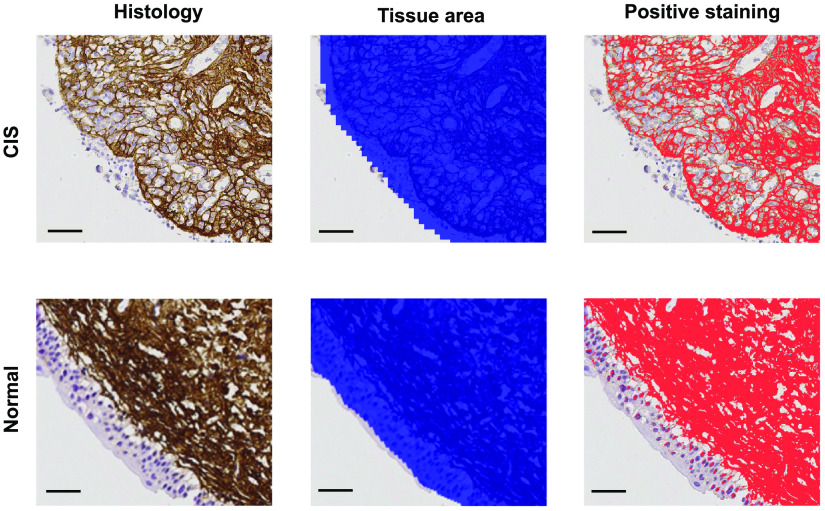
IHC positive staining area analysis. Representative staining results on CIS and normal sample are shown on the top and bottom row, respectively. For each IHC histology image, the total tissue area (blue) and the positive staining area (red) were determined for a given ROI. The positive staining ratio is calculated from these two measurements. Scale bar=50  μm.

Although no differences in staining scores were observed in the IHC images of tumor versus benign samples (all slides have a score of 3+), our extended analysis of the positive staining area compared with the total tissue area in the region of interest revealed that CIS samples show the lowest percentage of positive staining area: in brief, breakdown of collagen fibrils and architecture and/or bundling of collagen fibers led to a more “porous” submucosal layer (Fig. 8). Type I collagen is the most abundant extracellular matrix (ECM) component in the LP of the bladder, and its remodeling during tumor development has been well studied in many soft organs.^30^^,^^31^^,^^66^^,^^70^ Degradation of ECM and deposition of a new ECM that favors tumor growth are the major events of the remodeling process.^71^^,^^72^ Excessive remodeling of the ECM is characteristic of malignancy in many tumor types and has been shown to have strong correlation with tumor progression and poor prognosis.^66^^,^^67^^,^^73^^–^^76^ Changes in the morphology and organization of collagen fibers has been studied in non-muscle-invasive bladder cancer research: *ex vivo* Ta/T1 and CIS bladder samples were found to have straighter collagen fibers in the LP than healthy samples, which have regularly organized, wavy collagen fibers. That study concluded that more densely organized, straighter collagen is correlated with muscle-invasive progression, with dense collagen deposition likely preceding muscle invasion and facilitating tumor cell proliferation.^31^ For many samples in their study, both degradation and deposition events in the remodeling process had already taken place. In contrast, we limited our tumor samples to CIS, an early-stage tumor with cancer cells confined to the urothelium. We also observed changes in collagen morphology, in which the fibers lost their waviness and well-organized characteristics and became thinner, fragmented, and loosely organized. Such changes are responsible for the values obtained with MultiPIPE: fragmentation and loss of alignment in CIS make its LP layer less birefringent; similarly, thinning and loss of anisotropy reduce the AC of the LP layer. Although no studies to date have described the exact biological events occurring at the microscopic level during CIS tumor development in the bladder, we hypothesize that the morphological changes that we observed for this high-grade tumor belong to the degradation event of ECM remodeling that precedes actual tumor invasion into the LP layer.^77^^,^^78^ Relatedly, other concurrent biological changes, including edema and growth of new vasculature in the submucosa of CIS samples, could have also contributed to collagen reorganization.^14^^,^^79^ Inflamed samples in our study also exhibit some degree of collagen remodeling and do not have the same morphology as benign samples (i.e., straighter, denser fiber alignment near lymphoid aggregates). Notably, we did not observe the same level of collagen fragmentation and loss of anisotropy in the inflamed samples as in CIS.

The changes that we observed in the AC measurement of the urothelium in CIS samples can be explained by its high-grade nature. As high-grade tumor cells are histologically characterized by their hyperchromatic pleomorphic nuclei and a decrease in the cytoplasmic–nuclear ratio, tumor cells scatter light more than normal cells.^49^^,^^80^ Previous literature has described OCT images of CIS samples as losing delineation between the urothelium and LP layers, presented as smaller differences in the scattering properties of these two layers.^65^^,^^81^ In support of this finding, in our study, the measured AC ratio (U to LP) from CIS samples approached unity, which could stem from a combination of changes in both the urothelium and the LP layers of CIS tissue: first, the high-grade tumor cell nuclei become hyperchromatic and enlarged and therefore absorb and scatter light more, leading to an increase in the ${\mathrm{AC}}_{U}$ measurements; next, the degrading collagen in the LP region due to tumor development leads to reduced scattering and consequently a decrease of the ${\mathrm{AC}}_{\mathrm{LP}}$.

## 4 Conclusion

Taken together, the novel combination of quantitative birefringence^82^ and light attenuation metrics generated by MultiPIPE imaging is highly specific for differentiating CIS from benign tissue—including inflammation—in fresh human bladder biopsies and demonstrates, for the first time, the power of multiparameter, quantitative models in improving the specificity of bladder cancer diagnosis. Importantly, the MultiPIPE measurements correlate well with changes of morphological features in histological assessments that are used to diagnose cancer, lending credibility to their truth.

In addition to the specificity advantage, MultiPIPE imaging carries an important advantage over other surface-viewing modalities such as CLE, which are limited to viewing ∼100 to 150  μm into the tissue; hence, these technologies cannot extract a sufficient signal from the LP, which is the layer that contributes most to MultiPIPE analysis. In addition, the correlation study of MultiPIPE imaging parameters with morphological features as well as the image pairs shown in Fig. 5 demonstrates the value of MultiPIPE imaging for *in vivo* use, given the high correlation with histology and the ability to create histology-like cross-sectional images with relevant biological information quickly and non-invasively. Although our study was performed *ex vivo*, numerous investigations have proven that polarization-sensitive OCT, the technology used to generate the MultiPIPE datasets, can be implemented *in vivo*.^32^^,^^83^^,^^84^ Miniaturization strategies of OCT and polarization-sensitive OCT systems have been introduced in recent studies,^85^^,^^86^ suggesting the clinical feasibility of the integration of proposed analysis with both rigid and flexible cystoscopy systems during surveillance. Given this, MultiPIPE imaging is well poised for clinical translation and can serve as a critical technology to aid in effective detection and eradication of UBC. The meaningful results presented in this study, which derive from a rich dataset per sample subjected to rigorous statistical methods and blinded assessment despite its limited sample size, suggest a strong benefit to a larger, multicenter study to collect and validate MultiPIPE analysis of *in vivo* data. In addition, a future study on the tumor microenvironment during CIS development and progression would provide insights on the biological nature of our findings and reveal other biomarkers for correlation with the MultiPIPE metrics. The quantitative nature of our study allows for future development of robust diagnostic models that aid objective, accurate clinical decisions in real time, which will ultimately benefit patients by reducing the number of unnecessary procedures and shortening operating room time, leading to more timely and accurate treatment.

## Code, Data, and Material Availability

Data and code developed in this study are available upon reasonable request to the corresponding author.
